# Theoretical analysis of MWCNT and MXene/High-k pH BioFET sensors for biomedical applications

**DOI:** 10.1038/s41598-025-19805-x

**Published:** 2025-10-15

**Authors:** Gaurav Dhiman, Abhinandan Routray

**Affiliations:** 1 School of Computing, DIT University, Dehradun, Uttarakhand 248009 India; 2 Department of Electrical and Electronics Engineering, Manipal Institute of Technology, Manipal Academy of Higher Education, Manipal, Karnataka 576104 India

**Keywords:** PH, Si/SiO_2_, BioFET, MXene/High-k, MWCNT, Engineering, Materials science, Nanoscience and technology

## Abstract

In this paper, a theoretical analysis and comparative study of pH BioFET sensor designs using silicon/silicon dioxide (Si/SiO_2_), multi-walled carbon nanotube (MWCNT), and MXene/High-k dielectric materials is conducted. Being able to measure pH accurately and quickly is crucial in various fields, like clinical diagnostics, environmental study, food quality control, and industrial process management. Traditional sensing methods often have limitations in terms of sensitivity, selectivity, portability, and continuous observing. The incorporation of advanced materials, specifically carbon nanotubes and Ti_3_C_2_Tx MXene, into biosensor designs leads to improved sensing capacities, primarily attributed to their elevated surface-to-volume ratios, heightened sensitivity, and inherent biocompatibility. The study details the structural aspects of each BioFET device and employs a mathematical model to analyze their performance. Simulation results demonstrate that the MXene/High-k device exhibits superior electrical properties, including higher drain current, and transduction sensitivity, making it highly promising for advanced pH sensing applications compared to conventional Si/SiO_2_ and MWCNT-based sensors.

## Introduction

Sensors capable of detecting pH are crucial for diverse applications, ranging from clinical diagnostics, environmental monitoring to food quality control, and industrial process management. The ability to accurately and rapidly measure these parameters is paramount for effective disease management, ensuring public health, and maintaining ecological balance. Traditional sensing methods often face limitations concerning sensitivity, selectivity, portability, and real-time monitoring capabilities. This has driven extensive research into novel materials and fabrication techniques for developing advanced sensor platforms^1,2^. Wearable biosensors made from field effect transistors for pH monitoring is an emerging field. The FET based biosensors are known for their sensitivity, versatility, and selectivity and wide range of detection possibilities. The fabrication of FETs is well established process, and it is cost effective also^3,4^.

The incorporation of new materials such as carbon nanotubes (CNTs) in biosensors provides a foundation for highly sensitive and conductive environment for detecting various analytes in sweat with their remarkable characteristics such as high surface to volume ratios, good sensitivity, biocompatibility, energy efficiency, and relatively easier fabrication^5^.

To boost the sensing capabilities of bio-sensing devices, the paper proposes incorporation of Ti_3_C_2_Tx MXene as channel material for BioFET. These two-dimensional transition metal carbides significantly enhance the performance of FET-based sensors. When added to the channel material, Ti_3_C_2_Tx MXenes improve electronic properties, increase reactivity, and elevate sensitivity due to their high surface area. Furthermore, their biocompatibility and catalytic role accelerate sensor response. The findings demonstrate that composites featuring Ti_3_C_2_Tx MXenes notably improve the performance of wearable sensors^6,7^.

Non-enzymatic sensors have attracted significant attention owing to their low cost, high stability, rapid response, and excellent low limit of detection (LOD). However, their sensitivity and selectivity still lag enzymatic counterparts. While invasive monitoring remains the conventional practice, non-invasive sensing is increasingly regarded as a more promising strategy for continuous, real-time monitoring of blood analytes. Moreover, valuable diagnostic information can also be extracted from alternative body fluids such as sweat, saliva, tears, and interstitial fluid. Based on their underlying detection principles, biosensors are generally classified into three main categories: electrochemical, optical, and bio-electronic^8^. The details of the bio-electronic BioFET pH sensor used in this work are discussed in the following sections.

### Traditional Si/SiO_2_ based sensors

Silicon and its native oxide, silicon dioxide, form the cornerstone of modern semiconductor technology. The well-established fabrication processes and excellent material properties make Si/SiO_2_ an attractive platform for developing highly integrated and miniaturized sensors^9^.

Ion-Sensitive Field-Effect Transistors (ISFETs) are a prominent example of Si/SiO_2_ based pH sensors. In an ISFET, the gate metal of a conventional MOSFET is replaced by a pH-sensitive dielectric layer that is exposed to the analyte solution. The surface potential at the dielectric/electrolyte interface changes with pH, modulating the channel current of the transistor^10^. The Si/SiO_2_ layer, being the native oxide of silicon, offers good biocompatibility and can be easily integrated into standard CMOS fabrication processes. Researchers have explored various modifications to the Si/SiO_2_ surface to improve sensitivity, stability, and selectivity. For instance, modifying the Si/SiO_2_ surface with different functional groups or doping it with specific ions can enhance its affinity for H + or OH− ions, leading to improved pH response. Despite the advantages of Si/SiO_2_ in terms of miniaturization and cost-effectiveness, challenges related to long-term stability, drift, and calibration still need to be addressed for widespread clinical applications^11^.

### Multi-walled carbon nanotube (MWCNT) based sensors

Composed of multiple concentric graphene layers, Multi-walled carbon nanotubes (MWCNTs) are cylindrical nanostructures. Their unique combination of exceptional electrical conductivity, high aspect ratio, large surface area, mechanical strength, and chemical stability makes them perfect for a wide range of sensing applications. The MWCNTs can be readily functionalized with various chemical groups or biological recognition elements, significantly improving their sensing performance^12^.

MWCNTs exhibit inherent sensitivity to pH changes due to the presence of surface defects and functional groups (e.g., carboxyl, hydroxyl) that can protonate or deprotonate in response to variations in H^+^ ion concentration. This change in protonation state alters the charge carrier concentration and, consequently, the electrical resistance or capacitance of the MWCNT network, forming the basis for pH sensing. MWCNT-based potentiometric and impedimetric pH sensors have been developed, offering advantages such as fast response times and good stability. Furthermore, MWCNTs can serve as excellent electrode materials for immobilizing pH-sensitive dyes or conducting polymers, leading to composite sensors with enhanced performance^6,13–15^.

### MXene/high-k dielectric based sensors

MXenes are a new family of two-dimensional (2D) transition metal carbides, nitrides, and carbonitrides, characterized by the general formula M_{n+1}_X_n_T_x_, where ‘M’ is an early transition metal, ‘X’ is carbon or nitrogen, and ‘T_x’_ represents surface terminations (e.g., –OH, –O, –F). Their unique properties, including high metallic conductivity, large surface area, tunable surface chemistry, and hydrophilicity, make them exceptional candidates for electrochemical sensing. High-k dielectrics (materials with high dielectric constants) are often integrated with MXenes to form heterostructures that can further enhance sensor performance by improving gate control and reducing leakage currents^16^. The abundant surface termination groups on MXenes can interact with ions in solution, making them inherently sensitive to pH changes. The layered structure and excellent electrical conductivity of MXenes allow for efficient charge transfer, which is crucial for potentiometric or impedimetric pH sensing. Integrating MXenes with high-k dielectrics can lead to the development of highly stable and sensitive pH sensors by leveraging the superior gate coupling provided by the high-k material. While research on direct MXene/High-k pH sensors is emerging, the principles of MXene’s pH sensitivity, coupled with the enhanced dielectric properties of high-k materials, suggest a promising avenue for advanced pH sensing devices with improved signal-to-noise ratios and stability in complex biological environments^17,18^.

### Other bio-electronic pH sensors

Among the various dielectric/channel materials reported for ISFET pH sensors, oxides such as SnO_2_, Ta_2_O_5_, and sol–gel TiO_2_ are widely recognized for their stability and near-Nernstian performance^19–21^. These remain the dominant choices in many biomedical and chemical sensing studies. Further, pH monitoring is not only important in chemical and environmental contexts but also plays a critical role in bio-medical diagnostics. For instance, maintaining blood pH within the narrow physiological range of 7.35–7.45 is essential for homeostasis, and even small deviations may indicate conditions such as acidosis or alkalosis. Similarly, saliva pH (typically 6.2–7.6) is increasingly recognized as a non-invasive marker for oral and systemic health, while interstitial fluid (ISF) provides valuable information for wearable biosensors in personalized healthcare. These biological matrices, however, present unique challenges compared to standard buffer solutions, including protein adsorption, biofouling, high ionic strength, and viscosity, all of which can interfere with accurate pH measurement. Therefore, sensor materials designed for biomedical applications must combine high sensitivity with stability, robustness against interference, and compatibility with complex fluids. In this context, MXene based high-*k* dielectric structures offer strong potential due to their large surface area, hydrophilic nature, and superior charge-transport properties, which may support reliable operation even under bio-relevant conditions.

In the present work, however, our comparisons are focused on Si/SiO_2_ and MWCNT-based structures. Si/SiO_2_ represents the most classical ISFET configuration and serves as a useful baseline reference to highlight the improvements achieved with alternative materials. On the other hand, MWCNTs are an important class of carbon nanomaterials with excellent conductivity and surface properties and thus provide a relevant nanomaterial benchmark. By comparing our MXene/High-k with both a traditional (Si/SiO_2_) and a nanomaterial-based (MWCNT) reference, we demonstrate the advancement of our approach across two important categories of ISFET pH sensors, while also acknowledging the established contributions of oxide-based devices.

## BioFET structural details

This section breaks down each of the three material profiles defined in the model, explaining the rationale behind their characteristic parameters.

### Device 1: standard silicon (Si/SiO_2_) BioFET

**Fig. 1:**
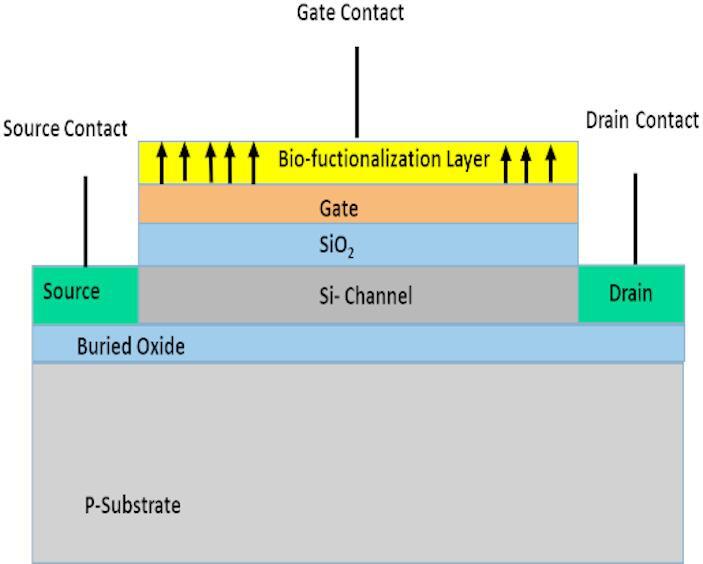
BioFET sensor design using Si/SiO_2_.

Figure 1 represents the most traditional and well-established BioFET architecture. It is based on a standard *n*-channel Metal-Oxide-Semiconductor Field-Effect Transistor (MOSFET) built on a silicon substrate with a silicon dioxide (SiO_2_) gate dielectric. The sensing occurs when charged biomolecules at the SiO_2_ surface modulate the electric field in the silicon channel. The typical threshold voltage for a standard *n*-channel enhancement-mode silicon is around 0.5 V, which requires a positive gate voltage to turn on.

### Device 2: proposed MWCNT BioFET

**Fig. 2:**
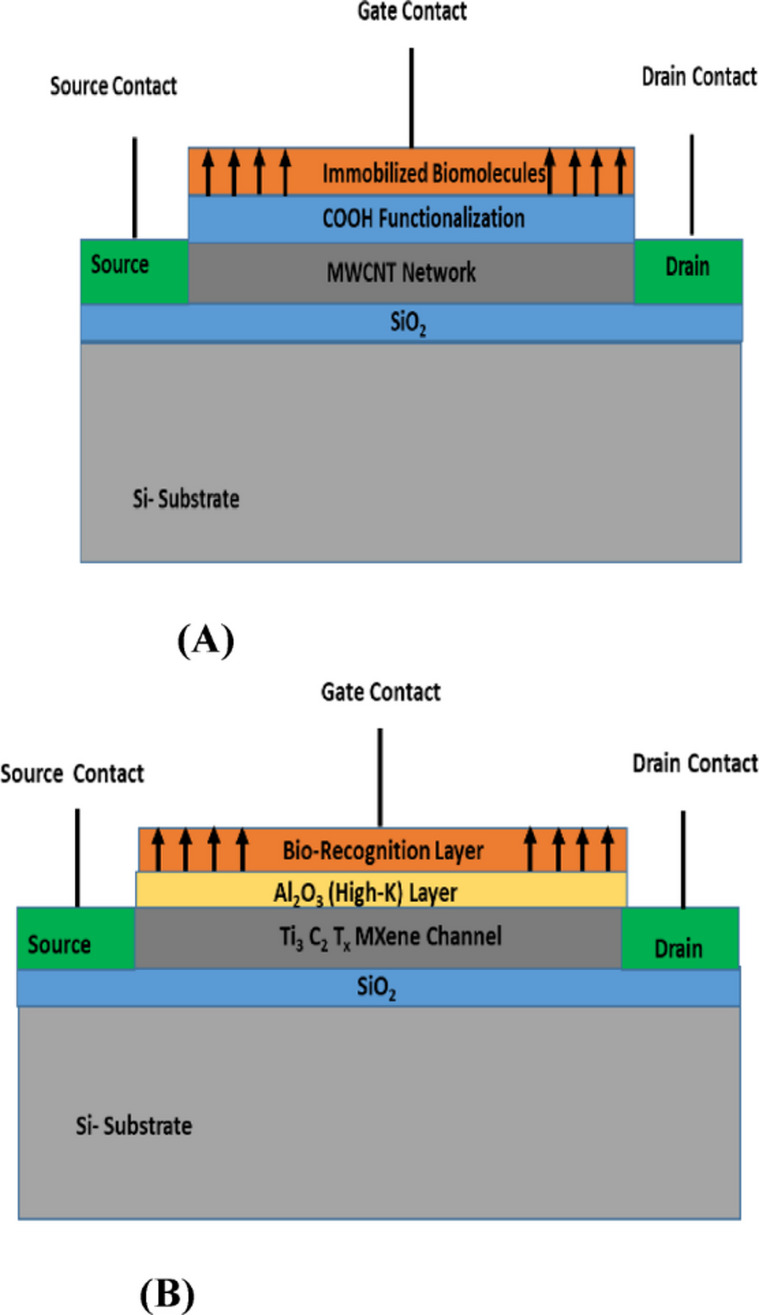
Proposed BioFET sensor designs. (**A**) MWCNT/SiO_2_. (**B**) MXene/High-k.

Figure 2A represents a BioFET where the channel is formed by a network of multi-walled carbon nanotubes (MWCNTs) deposited on a standard SiO_2_ dielectric. This proposed FET sensor are front gate devices made on silicon substrate with SiO_2_ layer. The channel between source and drain are made up of MWCNT.

### Device 3: proposed MXene BioFET

Proposed MXene BioFET shown in Fig. 2B models a next-generation BioFET using a 2D material, specifically MXene (Ti_3_C_2_T_x_), as the channel. Due to the sensitivity of MXenes to oxidation, a protective and functional high-k dielectric layer of Al_2_O_3_ is used on top. These proposed FET sensors are front gate devices made on silicon substrate with SiO_2_ layer. The bio-recognition layer acts as a gate for detecting variations in pH and other bio-sensitive analytes, including ions, proteins, enzymes, and nucleic acids, thereby enabling selective and sensitive biochemical sensing.

## Mathematical model of BioFET sensor

In 1970, P. Bergveld introduced the ion-sensitive FET (ISFET), a novel device derived from the conventional metal-oxide-semiconductor FET (MOSFET) where a liquid gate replaced the traditional metal gate electrode^22^. The ISFET can be transformed into a bio-FET by functionalizing its sensing surface with specific receptor molecules. The binding of charged target molecules to these receptors modulates the potential distribution and electrostatic interaction within the device. For a conventional MOSFET operating in the linear region, the drain-to-source current (*I*_ds_) is described by the following equation:

1 $$\:{I}_{\text{d}\text{s}}\:=\:{C}_{\text{O}\text{X}}{{\mu\:}}_{\text{r}\text{e}\text{f}}\:\frac{W}{L}\left[\right({V}_{\text{g}\text{s}}\:-\:{V}_{\text{t}0})\:{V}_{\text{d}\text{s}}\:-\:\frac{1}{2}{V}_{\text{d}\text{s}}^{2}]$$

For saturation region, *I*_ds_ is given as:

2 $$\:{I}_{\text{d}\text{s}}\:=\:{C}_{\text{O}\text{X}}{{\mu\:}}_{\text{r}\text{e}\text{f}}\:\frac{W}{2L}{\left({V}_{\text{g}\text{s}}\:-\:{V}_{\text{t}0}\right)}^{2}(1+{\uplambda\:}{V}_{\text{d}\text{s}}\text{})$$

Here, *C*_OX_ denotes the insulator capacitance per unit area, *µ*_ref_ is the carrier mobility within the channel, $\:V$_gs_ and $\:V$_ds_ are the applied gate and drain voltages, and *L* and *W* represent the length and width of the FET, respectively. $\:{\uplambda\:}$ is channel modulation parameter. For the Si/SiO_2_ Bio-FET, the threshold voltage is calculated using the classical MOSFET equation as follows:

3 $$\:{V}_{\text{t}0,\text{r}\text{e}\text{f}}\:=\:\frac{{\varPhi\:}_{ms}}{q}-\:\frac{{Q}_{\text{O}\text{X}}\:+\:{Q}_{\text{S}\text{S}}\:+\:{Q}_{\text{B}}}{{C}_{\text{O}\text{X}}}\:+\:2{\varPhi\:}_{\text{f}}$$

Here, $\:{\varPhi\:}_{ms}$ signifies the work function difference between the metal ($\:{\varPhi\:}_{m}$) and the semiconductor ($\:{\varPhi\:}$s). *Q*_OX_, *Q*_SS_, and *Q*_B_ denote the charge in the oxide layer, the charge at the oxide-semiconductor interface, and the depletion charge within the semiconductor, respectively. $\:{\varPhi\:}_{f}$ represents the Fermi potential. The values of $\:{\varPhi\:}_{ms}$ ≈ − 0.75 V, 2$\:{\varPhi\:}_{f}$ ≈ 0.87 V, *Q*_B_ ≈ 2.4 × 10^−7^ C/cm^2^, *Q*_OX_ ≈ 1.0 × 10⁻8 C/cm^2^ and *Q*_ss_ ≈ 4.8 × 10^−5^ C/cm^2^ are used in the calculations. The resulting threshold voltage is close to the 0.5 V used in our simulations.

For advanced channel materials (MXene and MWCNT), the threshold voltage expression simplifies to:

4 $${V_{{\text{t}}0{\text{ref}}}}\,=\,{\varPhi_{{\text{ms}}}} - {\text{ }}\left( {{Q_{{\text{ss}}}}/{C_{{\text{ox}}}}} \right)$$

since bulk-related terms (2$\:{\varPhi\:}_{f}$, *Q*_B_) are not directly applicable. The threshold voltage in these cases is mainly governed by the work function difference ($\:{\varPhi\:}_{ms}$) and interface charges (*Q*_ss_), with values consistent with experimental reports on these materials. For a bio-FET, since the gate is exposed to a solution and the gate voltage is applied via a reference electrode, its threshold voltage (*V*_t0, eff_) is given below as:

5 $$\:{V}_{\text{t}0,\:\:\text{e}\text{f}\text{f}}\:=\:{V}_{\text{t}0,\text{r}\text{e}\text{f}}\:+\:{\varDelta\:V}_{\text{t}0}$$

where $\:{\varDelta\:\text{V}}_{t0}$ is change in threshold voltage induced by bio sensing material is given by:

6 $$\:{\varDelta\:V}_{\text{t}0}=\:{\delta\:}_{\text{p}\text{H}}(\text{p}\text{H}-{\text{p}\text{H}}_{\text{r}\text{e}\text{f}})$$

where $\:{\delta\:}_{pH}$ is intrinsic chemical sensitivity (assumed to be 0.05 V/pH for all devices). pH and pH_ref_ are pH value of material and reference pH value. The parameter δpH is an intrinsic property of the sensing dielectric surface (e.g., SiO_2_, Al_2_O_3_, HfO_2_), rather than the semiconductor channel material (Si, MXene, MWCNT). In our comparative modeling framework, we assumed the same intrinsic chemical sensitivity across the devices in order to isolate and highlight the effect of different channel materials on the overall electrical transduction process. This ensures a fair comparison and the gate dielectric responds equally to pH changes, while differences in the device output arise from the channel properties and electrostatics. The model for drain current incorporates significant factors, one of which involves subthreshold conduction, temperature effects, noise effects and mobility degradation. Table 1 gives the details of parameters used for the model and their respective values for three devices.

**Table 1:** Parameters used in comparative analysis.

Parameter name	Symbols	Si/SiO_2_	MXene/High-k	MWCNT/SiO_2_
Threshold voltage reference	*V*_t0ref_	0.5 V	− 0.2 V	0.1 V
Electron mobility	*µ*_ref_	600 cm^2^/Vs	2000 cm^2^/Vs	500 cm^2^/Vs
Subthreshold slope	SS	80 mV/decade	100 mV/decade	90 mV/decade
Channel length modulation parameter	*λ*	0.01 1/V	0.05 1/V	0.15 1/V
Oxide thickness	*t*_ox_	5 nm	5 nm	10 nm
Channel length	*L*	180 nm	180 nm	180 nm
Aspect ratio	*W/L*	10	10	10
Work function difference	*Φ*_ms_ (V)	− 0.049 eV	− 0.1	+ 0.19
Fermi potential	2*Φ*_f_ (V)	0.60 V	–	–
Depletion charge	*Q*_B_ (C/cm^2^)	2 × 10^− 8^	–	–
Oxide charge	*Q*_*OX*_(C/cm^2^)	1.0 × 10^− 8^	–	–
Interface charge	*Q*_ss_ (C/cm^2^)	5 × 10⁻^9^	3.08 × 10^− 7^	3.11 × 10^−8^
Oxide capacitance	*C*_ox_ (F/cm^2^)	6.9 × 10^−7^	1.59 × 10^− 6^	3.45 × 10^−7^

## Fabrication feasibility of the proposed MWCNT and MXene-based BioFET

MWCNT fabrication is the combination of bottom-up material deposition with top-down lithography. First start with a Si/SiO_2_ substrate. The SiO_2_ surface may be pre-treated to promote CNT adhesion. The process of dispersion is used to disperse MWCNTs in a solvent of deionized water with a sodium dodecyl sulfate surfactant. Then deposit the MWCNT solution onto the substrate via drop-casting or spin-coating to form a random network. The photolithography process is used to define the source and drain electrodes. Deposition of metal (Ti/Au) contacts that have good adhesion to carbon is used for creating the metal contacts. The area of the MWCNT network between the source and drain electrodes automatically becomes the transistor channel. Excess MWCNTs outside this area can be removed with a plasma etch if required. With passivation process a protective layer is deposited, leaving the CNT channel exposed for sensing. The method of acid treatment (with H_2_SO_4_/HNO_3_) is used to create carboxyl (–COOH) groups on the nanotube surfaces, which can then be used for covalent linking of probes. This helps to enable the probe attachment to achieve the bio-functionality of MWCNTs^23,24^.

MXene fabrication process involves a bottom-up approach for the channel material combined with top-down lithography. The process starts with a base substrate, typically a heavily doped Si wafer with a layer of SiO_2_ (Si/SiO_2_). With etch process, aluminium (Al) layer is etched from a MAX phase precursor (e.g., Ti_3_AlC_2_) using an acid like HF or HCl/LiF. Then we delaminate the resulting multilayered MXene into single or few-layer flakes in solution, often using sonication. The MXene channel is formed by the process of deposition. The MXene flakes from the solution is deposited onto the substrate using methods like drop-casting or spin-coating. The process of photolithography is used to define the source and drain contact areas. Deposition of metal (Au) contacts is done. The lift of process is used to remove unwanted metal. The another process of lithography can be used to remove excess MXene layer. The native surface terminations (–O, –OH, –F) on MXenes are highly suitable for direct covalent bonding of biomolecules, often simplifying the functionalization process compared to silicon^25–28^.

## Results and discussions

**Fig. 3:**
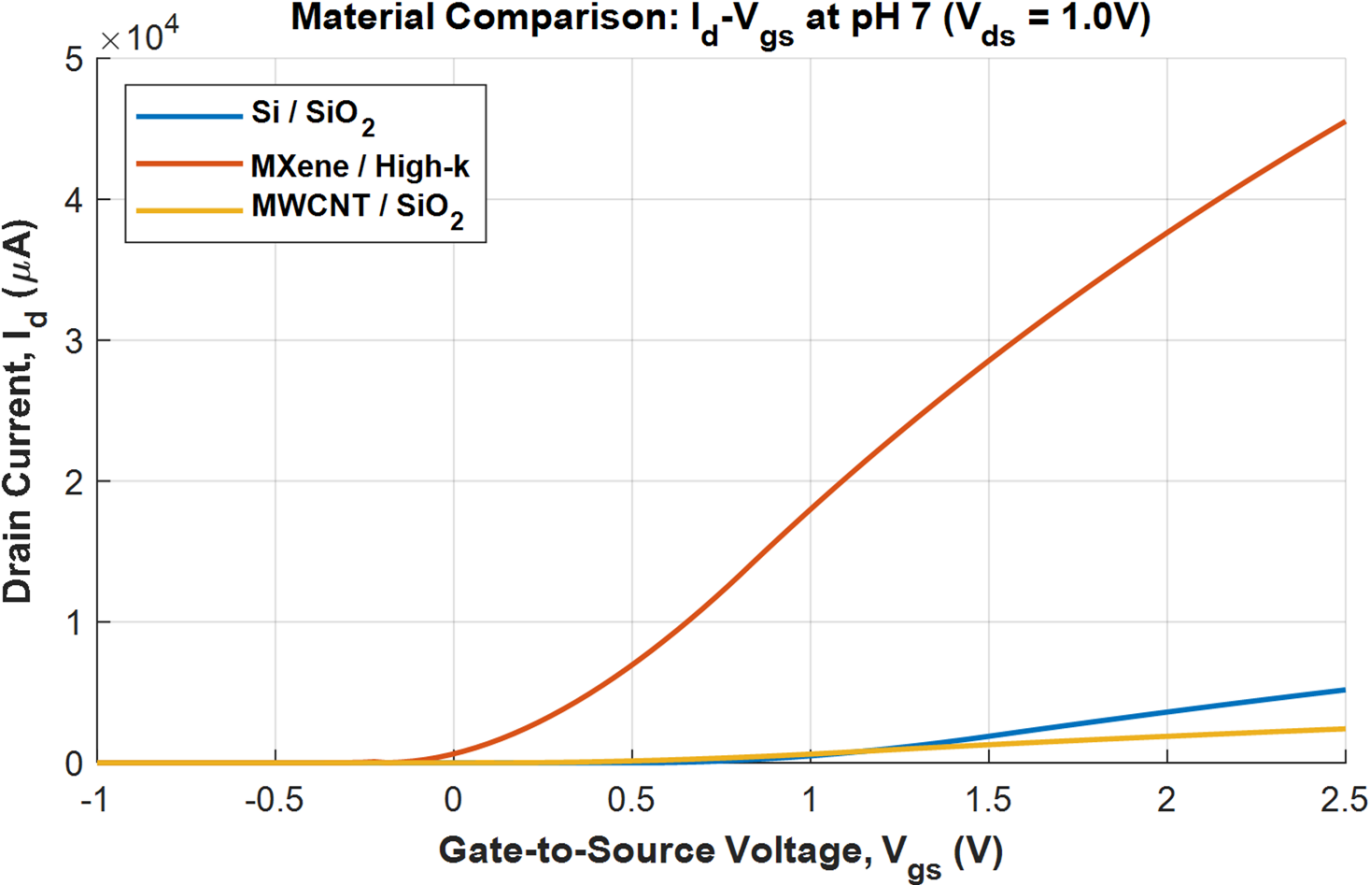
Transfer characteristics (*I*_d_-*V*_gs_).

The transfer characteristics drawn between drain current and gate to source voltage (*I*_d_–*V*_gs_) is shown in Fig. 3, provide a fundamental comparison of the three device profiles at a baseline (pH 7). The drain to source voltage (*V*_ds_) is kept at 1 V. The *V*_gs_ is varied from − 1 V to 2.5 V. MXene/High-k device exhibits the highest drain current (*I*_d_) for a given gate voltage (*V*_gs_). This is a direct consequence of the high carrier mobility and high gate capacitance assigned in the model, both of which contribute to a large transconductance. The negative threshold voltage effect is also evident as the device begins to conduct current even at slightly negative *V*_gs_. The Si/SiO_2_ device shows moderate current levels. This represents the well-understood, standard performance of a traditional MOSFET. MWCNT/SiO_2_ device shows the lowest current. While the intrinsic mobility of a single nanotube is high, the model correctly captures the lower effective mobility of a network, resulting in a lower overall current drive compared to the other materials. Its turn-on voltage is very close to zero, consistent with a network containing metallic pathways. The MXene/High-k profile demonstrates the highest potential for generating a large electrical signal due to its superior electrical properties. A larger current allows for a larger absolute change upon sensing, which can improve the signal-to-noise ratio.

**Fig. 4:**
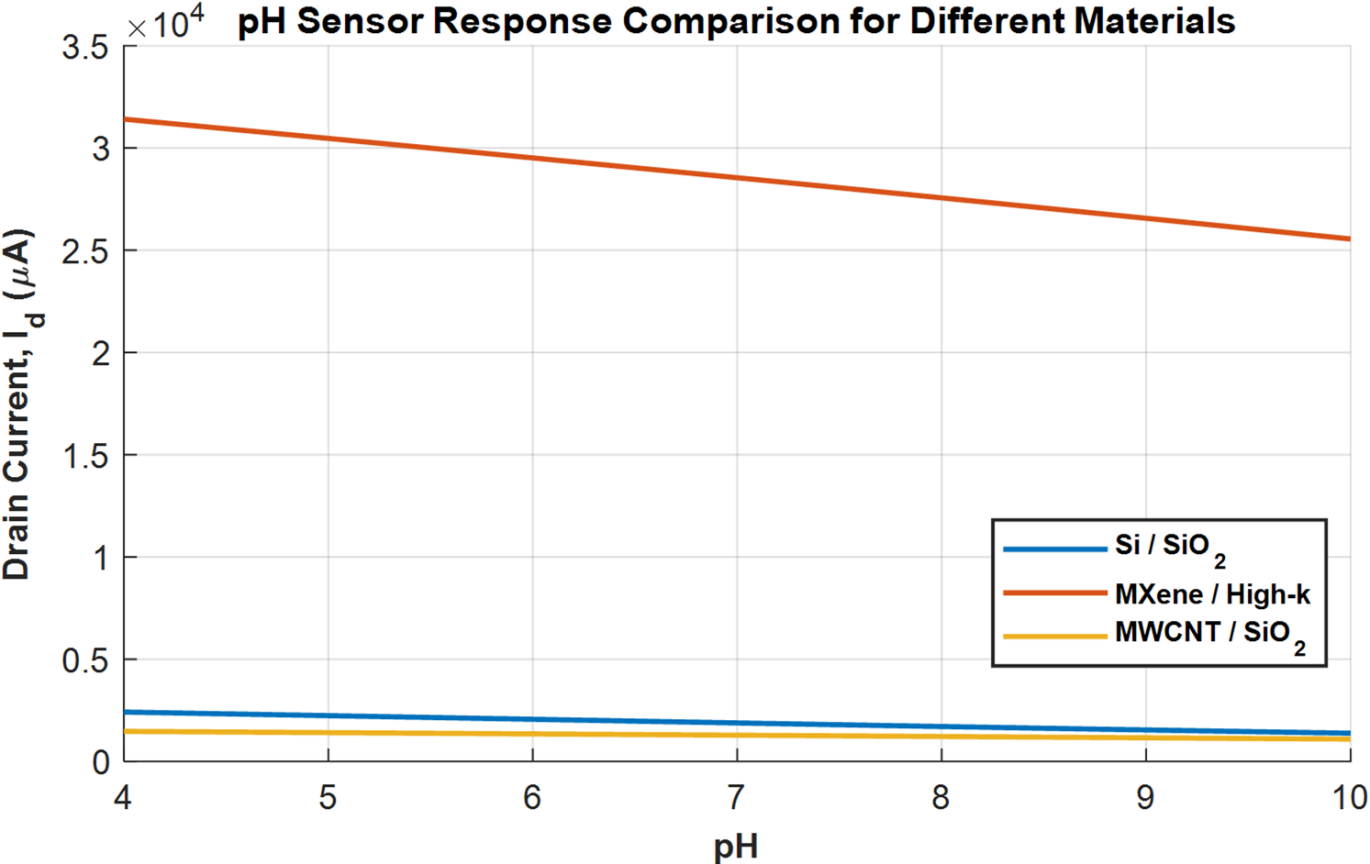
pH sensor response curve.

Figure 4 shows the drain current as a function of pH. The MXene/High-k device shows the largest absolute change in current across the pH range, again due to its high transconductance. A given change in threshold voltage induced by a pH change results in a larger change in drain current for a high-transconductance device. The Si/SiO_2_ and MWCNT/SiO_2_ devices show a smaller, but still clear, response.

**Fig. 5:**
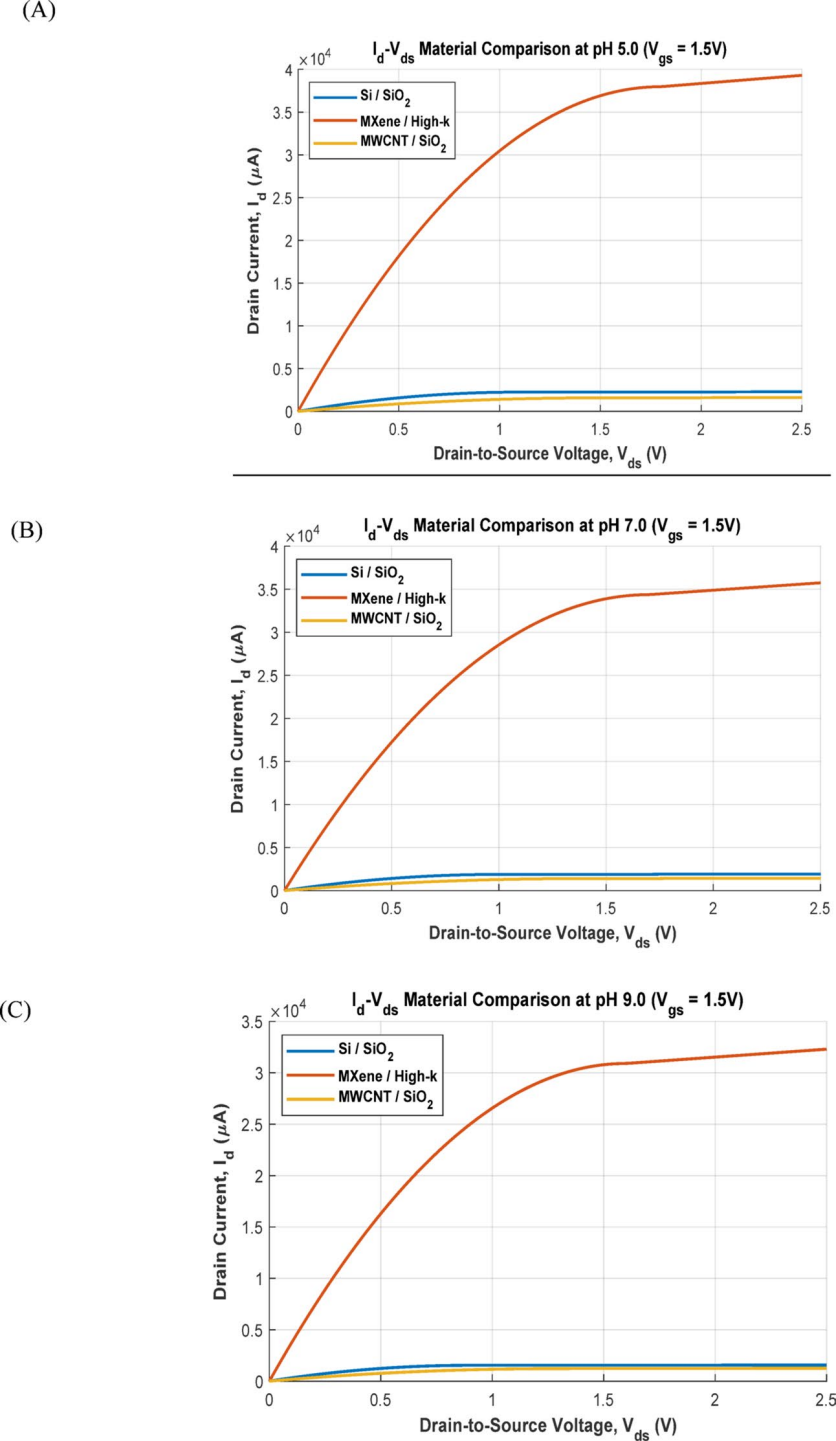
Output characteristics (*I*_d_ vs. *V*_ds_) at different pH. (**A**) pH = 5. (**B**) pH = 7. (**C**) pH = 9.

The *I*_d_-*V*_*d*s_ plots in Fig. 5A–C illustrate how the drain current is modulated by pH at a fixed gate voltage (*V*_gs_ = 1.5 V). The *V*_ds_ is swept between from 0 to 2.5 V. For each material, as the pH increases, the threshold voltage increases, leading to a lower drain current. The curves clearly show the linear and saturation regions of operation. The separation between the curves for different pH values is most pronounced for the MXene/High-k device, again indicating higher sensitivity. The Si/SiO_2_ and MWCNT/SiO_2_ devices also show clear modulation but with smaller current changes between pH steps.

**Fig. 6:**
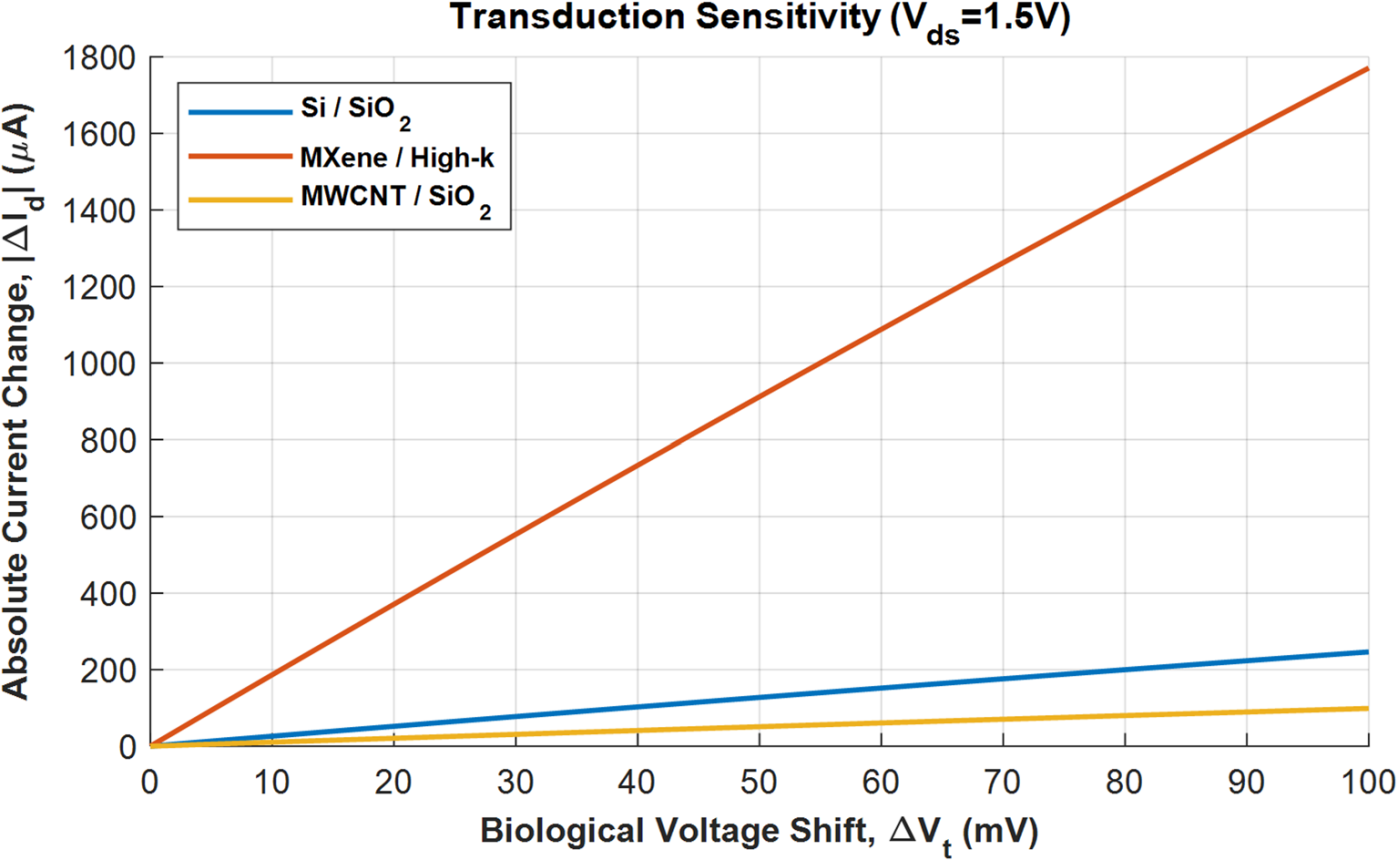
Transduction sensitivity for BioFETs.

The transduction sensitivity is shown in Fig. 6. It shows the ability of the transistor to convert the change in threshold voltage into a measurable drain current change is governed by its transconductance (*g*_m_), which is directly related to mobility and gate capacitance. The plot confirms that MXene/High-k device with higher mobility and gate capacitance produces a larger output signal for the same biological event, thereby offering higher sensitivity. While the MWCNT device shows lower current, its advantage in a real-world scenario would be its extremely high surface area, which could potentially lead to a larger number of binding events and thus a larger Δ*V*_t_ than modeled, partially compensating for its lower electrical performance. The Si/SiO_2_ device serves as the reliable, well-understood benchmark against which these newer materials are compared.

**Fig. 7:**
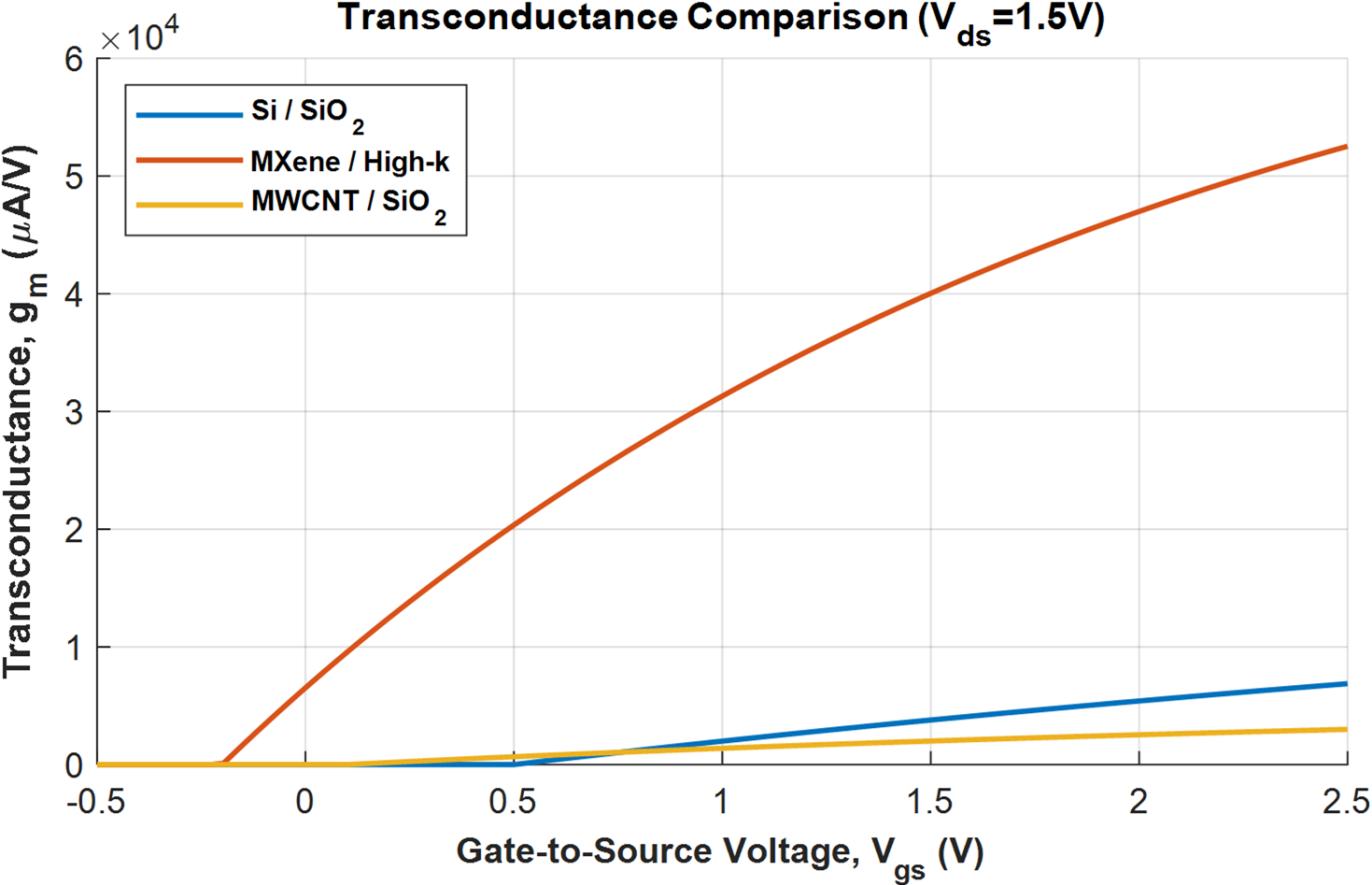
Transconductance vs. *V*_gs_ plot.

For pH sensing, the MXene device with the highest transconductance (as portrayed in Fig. 7) provides the highest current sensitivity. The simulation results consistently demonstrate a key principle of BioFET design that is sensitivity of the sensor is a product of both the biological interface and the electrical properties of the transistor.

**Table 2:** Comparative analysis with recent literature.

Materials	Sensing mechanism	Substrate	Sensitivity (mV pH- 1)	Detection range (pH)	RT (s)	Linearity	Ref.
Graphite	Electrochemical	Cellulose-polyester blend	4	6–9	5	–	^29^
Iridium oxide	Potentiometric	Ripstop silver	17.15	4–8	–		^30^
PEDOT: BTB	Chemo-resistive	Bio-ceramic fabric	8.3	2 to 7	–	0.997	^31^
CNT/polyaniline	Potentiometric	Waterproof polyester	45.9	5–9	–	–	^32^
ZnO	FET	polysilicon	− 59	1–9	–	–	^33^
HxWO3	Potentiometric	PET	− 53.6	2–8	10.5	–	^34^
PANI	Potentiometric	PEN	− 56.2	4–8	–	–	^35^
MXene/High-K	FET-potentiometric	Silicon	65.35	5–9	2.4	0.9984	Our work

Table 2 presents a valuable comparison of various pH BioFETs based on their core performance indicators. It provides a comparison of different pH sensor technologies. The sensitivity of a pH sensor, measured in millivolts per pH unit (mV/pH), is a critical metric for determining how well it responds to changes in pH. The MXene/High-K design, labeled demonstrates the highest sensitivity at 65.35 mV/pH. This value is significantly higher than most other entries and surpasses the theoretical Nernstian limit of approximately 59 mV pH at room temperature, suggesting a highly efficient transduction mechanism. Materials like Iridium Oxide (17.15 mV/pH)^30^, Graphite (4 mV/pH)^29^, and PEDOT: BTB (8.3 mV/pH)^31^ show notably lower sensitivities. The CNT/Polyaniline^32^ sensors have sensitivities of 60 mV/pH and 45.9 mV/pH respectively, which are also high but still below the MXene/High-K design. The ZnO^33^ and PANI^35^ sensors show negative sensitivity values (− 59 mV/pH and − 56.2 mV/pH), indicating a reverse trend in response. This is not unusual for pH sensors and shows that their sensitivity is close to the Nernstian ideal.

Response time (RT) is crucial for real-time monitoring applications, such as in clinical diagnostics or industrial processes. The MXene/High-K design stands out with the fastest response time recorded in the table at just 2.4 s. The Graphite sensor has a response time of 5 s, and HxWO_3_ has a response time of 10.5 s. Most other entries lack a reported response time, which makes a direct comparison difficult, but the MXene/High-K sensor’s speed is a significant advantage. Linearity is measured by the coefficient of determination (*R*^2^) and indicates how well the sensor’s response follows a straight line over its detection range. A value closer to 1 signifies more reliable and accurate measurements. The MXene/High-K sensor exhibits a very high linearity with an *R*^2^ value of 0.9984, suggesting excellent consistency and reliability within its detection range. The PEDOT: BTB sensor also has a high linearity of 0.997. Other entries do not report this value.

Finally, in light of the comparative analysis with recent literature (Table 2), the MXene/high-k design demonstrates promising near-Nernstian voltage sensitivity and competitive transduction efficiency relative to leading oxide and 2D-material pH sensors within comparable pH windows and operating conditions.

To clarify the positioning of our proposed device, it is important to compare the three commonly reported ISFET-based pH sensing approaches—potentiometric, amperometric, and impedancemetric—against the BioFET configuration used in this work. Potentiometric ISFETs remain the most widely adopted for pH monitoring due to their simplicity and direct transduction of proton concentration into electrical signals, although they are often limited by drift and Nernstian sensitivity. Amperometric sensors offer high current-based responses but depend on specific redox reactions and can suffer from electrode fouling, while impedancemetric sensors provide excellent sensitivity but are prone to noise and stability issues in complex media. As summarized in Table 3, our BioFET belongs to the potentiometric ISFET family but leverages MXene/MWCNT High-k dielectric materials to achieve enhanced sensitivity, lower detection limits, and improved robustness against interference. These features make the proposed device highly promising for real-time biomedical applications, particularly in sweat, saliva, and interstitial fluid monitoring.

**Table 3:** Comparison of performance indices of different sensors.

Sensor type	Working principle	Typical detection range	Sensitivity	Limit of detection (LOD)	Stability and challenges	Relevance to biomedical use
Potentiometric ISFET^36^	Measures gate potential change due to H⁺ ion concentration	pH 2–12 (reliable in ~ 6–8 clinically)	Near-Nernstian (~ 59 mV/pH)	~ 0.05–0.1 pH	Good, but subject to drift and biofouling	Widely used; simple, label-free, but can suffer interference
Amperometric^37^	Measures current from redox reactions at electrode surface	Narrow, depends on redox couple	High, but reaction-specific	~µM ion concentration equivalent	Affected by by-products and electrode fouling	Limited direct use for pH; more suited for metabolites
Impedancemetric^38^	Monitors impedance/capacitance change at electrode–electrolyte interface	Broad, but matrix-dependent	Very high (sub-Nernstian precision)	< 0.01 pH in optimized systems	Sensitive to noise and electrode polarization	Promising, but less mature for continuous pH monitoring
Proposed BioFET	Bioelectronic extension of potentiometric ISFET; channel current modulated by surface potential	pH 5–9(covers biomedical range ~ 6–8)	Enhanced (~ 65 mV/pH using MXene/MWCNT high-k dielectric)	< 0.05 pH (estimated)	Improved stability due to high-k dielectric and hydrophilic MXene; robust against interference	Highly relevant: non-invasive, patch-based pH monitoring in sweat, saliva, ISF

### Discussions

In this paper, a theoretical comparison of Si/SiO₂, MWCNT/SiO_2_, and MXene/High-k BioFETs is done, which provides useful predictive insights but also involves some limitations. The results presented here should be interpreted as predictive benchmarks that highlight material/channel-dependent trends rather than absolute performance values. The model assumes ideal material properties, uniform High-k dielectric behavior, and stable interfaces, while neglecting variability in material synthesis, device fabrication, and surface chemistry. Real-world devices often encounter non-ideal electrolyte/dielectric interactions, including drift, hysteresis, and charge trapping, which can reduce long-term stability. This work specifically targets biomedical applications by proposing BioFETs for non-invasive monitoring of pH biomarkers. The sensors are envisioned in patch-based formats for detecting pH in sweat or saliva, which are valuable indicators for real-time health monitoring and disease management. This work highlights the significance of accurately tracking these biomarkers and demonstrates how the proposed pH BioFETs can enhance measurement precision. Furthermore, the devices are designed using biocompatible materials, with the potential to be flexible, bendable, and disposable, making them suitable for practical biomedical deployment.

The present analysis demonstrates a generic pH-sensing framework that achieves both high sensitivity and stability, features highly relevant to biomedical applications. The observed response across pH 5–9 covers the clinically important range of ~ 6–8, enabling accurate detection of subtle variations in blood or interstitial fluid (ISF) that are diagnostically significant. Furthermore, the high capacitance of the MXene/MWCNT dielectric improves the signal-to-noise ratio, which is particularly beneficial in protein-rich or viscous environments such as serum or saliva, where conventional ISFETs often exhibit drift and reduced accuracy. These advantages suggest that the proposed sensing platform holds promise for clinical applications, including continuous blood monitoring in intensive care units, saliva-based point-of-care diagnostics, and wearable pH monitoring through ISF. Nonetheless, experimental validation in real biological matrices remains a limitation of this study. Addressing challenges such as biofouling, protein interference, and long-term stability will be an important direction for future work to fully establish the biomedical potential of this approach.

## Conclusions and future work

This paper has presented a comparative theoretical analysis of BioFET pH sensor designs based on Si/SiO₂, MWCNT/SiO₂, and MXene/High-k dielectric materials, highlighting their potential for bio-medical applications. The findings consistently demonstrate that the sensitivity of a BioFET sensor is a product of both the biological interface and the electrical properties of the transistor. The traditional Si/SiO₂ device serves as a reliable benchmark, leveraging well-established fabrication processes and good material properties for integrated and miniaturized sensors. However, challenges regarding long-term stability, drift, and calibration persist for widespread clinical use. MWCNT-based sensors, while exhibiting lower current drive in a network configuration, offer advantages due to their exceptional electrical conductivity, large surface area, and chemical stability, along with ease of functionalization for enhanced sensing capabilities. The proposed MXene/High-k device shows the most significant promise, demonstrating the highest drain current and transduction sensitivity. This superior performance is attributed to the high carrier mobility and gate capacitance of MXenes, which lead to a larger output signal for the same biological event, thereby improving the signal-to-noise ratio. The inherent sensitivity of MXenes to pH changes, coupled with the enhanced dielectric properties of high-k materials, positions them as excellent candidates for advanced pH sensing devices with improved stability in complex biological environments. The fabrication feasibility of both MWCNT and MXene-based BioFETs through combined bottom-up and top-down approaches further supports their practical implementation. This comparative study provides valuable insights for the design and development of next-generation BioFETs for accurate and real-time pH monitoring.

Overall, the present theoretical study should be regarded as a screening tool that highlights promising material/dielectric combinations. The encouraging results for the MXene/high-k configuration serve as motivation for further experimental exploration and optimization. The demonstrated superiority of MXene/High-k devices in the model represents theoretical potential, which will be experimentally validated focusing on fabricating prototype devices, and extending the modeling framework to incorporate reliability and interference factors as future study of this work. A combined theoretical-experimental approach will eventually be essential to validate the predicted advantages and establish the feasibility of these novel BioFET platforms for practical biochemical sensing applications.

## Data Availability

Yes, and this manuscript’s results are produced through mathematical modeling. The outputs of this modeling can be made available upon reasonable request once the manuscript is accepted. No supplementary datasets were generated or analyzed. Requests for data can be made to Gaurav Dhiman.
